# White-nose syndrome survivors do not exhibit frequent arousals associated with *Pseudogymnoascus destructans* infection

**DOI:** 10.1186/s12983-016-0143-3

**Published:** 2016-03-03

**Authors:** Thomas Mikael Lilley, Joseph Samuel Johnson, Lasse Ruokolainen, Elisabeth Jeannine Rogers, Cali Ann Wilson, Spencer Mead Schell, Kenneth Alan Field, DeeAnn Marie Reeder

**Affiliations:** Biology Department, Bucknell University, Lewisburg, PA 17837 USA; Department of Biosciences, Faculty of Biological and Environmental Science, Metapopulation Research Centre, University of Helsinki, Viikinkaari 1, Helsinki, Finland

**Keywords:** White-nose syndrome, *Myotis lucifugus*, *Pseudogymnoascus destructans*, Hibernation, Survival, Torpor, Periodic arousals

## Abstract

**Background:**

White-nose syndrome (WNS) has devastated bat populations in North America, with millions of bats dead. WNS is associated with physiological changes in hibernating bats, leading to increased arousals from hibernation and premature consumption of fat reserves. However, there is evidence of surviving populations of little brown myotis (*Myotis lucifugus*) close to where the fungus was first detected nearly ten years ago.

**Results:**

We examined the hibernation patterns of a surviving population of little brown myotis and compared them to patterns in populations before the arrival of WNS and populations at the peak of WNS mortality. Despite infection with *Pseudogymnoascus destructans*, the causative fungal agent, the remnant population displayed less frequent arousals from torpor and lower torpid body temperatures than bats that died from WNS during the peak of mortality. The hibernation patterns of the remnant population resembled pre-WNS patterns with some modifications.

**Conclusions:**

These data show that remnant populations of little brown myotis do not experience the increase in periodic arousals from hibernation typified by bats dying from WNS, despite the presence of the fungal pathogen on their skin. These patterns may reflect the use of colder hibernacula microclimates by WNS survivors, and/or may reflect differences in how these bats respond to the disease.

**Electronic supplementary material:**

The online version of this article (doi:10.1186/s12983-016-0143-3) contains supplementary material, which is available to authorized users.

## Background

White-nose syndrome (WNS) is an epizootic disease that has caused mass mortality in hibernating North American bats since 2006 [1]. The disease is caused by the psychrophilic fungus *Pseudogymnoascus destructans* (formerly known as *Geomyces destructans*, hereafter *Pd*), an ascomycete fungal pathogen [2]. *Pd* infection primarily affects bats during hibernation [3, 4] and fungal growth occurs optimally between 12–16 °C [5]. Since WNS was first documented in upstate New York in the winter of 2005–2006, it has spread rapidly across eastern North America and has been detected as far south as Mississippi (www.whitenosesyndrome.org). Population levels of highly susceptible species such as the little brown myotis (*Myotis lucifugus*) have plummeted in the areas affected by WNS, with predictions of regional extirpation in northeastern North America by 2026 [6, 7]. However, surviving populations of little brown myotis have been documented at sites where *Pd* was first detected nearly ten years ago [8], although the mechanisms that support survival are not known.

*Pd* is the first pathogen known to cause mortality in torpid mammalian hosts. Hibernating bats survive the energetic bottleneck of winter by building stores of body fat in late summer and early autumn and by conserving energy through extended torpor [9]. Because of restricted behavioral movements, extreme energy limitation, likely suppression of some immune responses [10, 11] and a narrow range of ambient temperature (*Ta*) in the hibernacula, torpid bats are susceptible hosts for pathogens that can tolerate low *Ta*, such as *Pd* [12]. *Pd* is invasive and damages the dermal tissue on the wings of bats during hibernation, forming characteristic cupping erosions that are diagnostic of the fungal infection [13]. These erosions cause inflammation [14] and lead to fluid and electrolyte loss across the damaged dermal tissue leading to a cascade of physiological disturbances [15].

Although the mechanism is still unknown, infection leads to increased arousals from torpor, resulting in the premature depletion of fat reserves and starvation [4, 16, 17]. Furthermore, increased arousals by conspecifics may lead to disturbances resulting in additional, unnecessary arousals [18]. Unaffected little brown myotis have been documented with an average torpor bout length (the number of days between euthermic arousals) of ca. 13 days [19], whereas little brown myotis dying of the infection have decreased torpor bout length to approximately 8 days [16]. Bouts of torpor are interrupted by periodic arousals to normothermy and the resumption of many physiological and behavioral processes [20]. The associated rewarming is energetically costly as is the brief normothermic resting period [21]. Although hibernating mammals spend less than 1 % of their time euthermic, they use up to 90 % of their stored energy during these periods [22, 23]. Therefore, survival of bats with WNS may be determined by the balance between the amount of fat stored and the number of arousals.

The function of arousal episodes during hibernation is still poorly understood and is likely a combination of several factors [23, 24]. It is unknown if the physiological responses needed for bats to defend against *Pd* infection occur at sufficient rates at the low body temperatures (T_b_) typical of a torpor bout during hibernation. Thus, periodic arousals may play an important role in the defense response of hibernating bats, as suggested for immune responses of hibernating ground squirrels [10]. Although periodic arousals are known to increase in frequency among bats afflicted with WNS, an increase in the duration of arousals has not been observed [4, 16, 25]. While increased frequency but not duration of arousals characterize bats that die of WNS, the thermoregulatory behavior of bats that survive WNS may be different and is unstudied. Maximal survival could be achieved by balancing different values of torpor bout length, arousal bout duration, body temperature during torpor and arousals, and microclimate selection [26].

To better understand the survival of free-ranging little brown myotis, we examined the hibernation patterns of a remnant population in New York and compared them to both pre-WNS hibernation patterns and patterns expressed in bats that died from WNS. We hypothesized that bats in remnant populations survive WNS despite an active *Pd* infection by displaying torpor and arousal behaviors that differ from the behavior of bats dying from WNS. We tested whether these behavioral population-level adaptations include changes in 1) arousal frequency, 2) arousal bout duration and skin temperature, and 3) microclimate selection reflected by skin temperature of bats in torpor. We also investigated the relationship between hibernation patterns and *Pd* infection intensity to gain insight into possible effects of these patterns on infection dynamics in bats that have survived WNS.

## Results

**Fig. 1:**
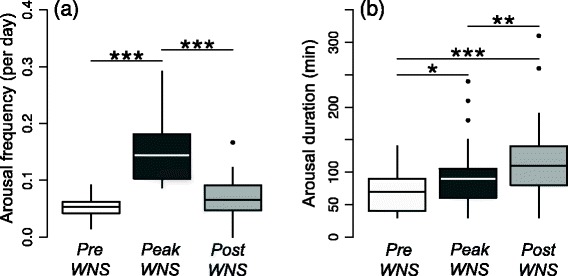
**a** Arousal frequency (arousals/day) in pre-WNS, peak-WNS and post-WNS bats. **b** Arousal duration in pre-WNS, peak-WNS and post-WNS bats. For statistical details, see Table 2. The asterisks ***, **, and * indicate statistical significance of *P* < 0.001, *P* < 0.01, and *P* < 0.05, respectively. Boxes depict the 25th and 75th percentiles, lines within boxes mark the median, and whiskers represent 95th and the 5th percentiles

Peak-WNS little brown myotis aroused significantly more frequently from torpor than either pre-WNS (*p* < 0.0001) or post-WNS bats (*p* = 0.0003; Tables 1 and 2, Fig. 1a). This is evident in their shorter torpor bout lengths (7.9 ± 5.5 days) as compared with pre-WNS (13.7 ± 9.0 days, *p* = 0.0060) or post-WNS bats (12.0 ± 10.8 days, *p* = 0.0184). The arousal frequency in post-WNS bats did not differ significantly from pre-WNS bats (*p* = 0.1773).

**Table 1:** Means, standard deviations (s.d.) and medians for the four response variables tested between study groups

		Arousal frequency (arousals/day)			Arousal duration (min)			Arousal Tsk (°C)			Torpor Tsk (°C)		
Group	n	Mean	SD	Median	Mean	SD	Median	Mean	SD	Median	Mean	SD	Median
pre-WNS	11	0.052	0.021	0.053	70	30	70	22.70	2.63	23.42	4.6	1.7	4.5
peak-WNS	12	0.15	0.063	0.14	86	38	90	24.52	2.73	24.84	7	1.2	7.3
post-WNS	19	0.068	0.039	0.066	116	50	110	22.73	2.54	23.06	1.9	1.4	1.6

**Table 2:** Model statistics for testing between-group differences in relative arousal frequency, duration (min) of arousal periods, arousal temperature and torpor temperature

Response	Comparison	Estimate	SE	*t*	*p*	Adjusted *p*
Arousal frequency	pre-WNS vs peak-WNS	−0.10	0.02	−5.27	<.0001	<.0001
	post-WNS vs peak-WNS	−0.09	0.02	−4.24	0.0001	0.0003
	post-WNS vs pre-WNS	0.02	0.01	1.42	0.16	0.1773
Arousal duration	pre-WNS vs peak-WNS	−0.23	0.11	−2.21	0.034	0.0445
	post-WNS vs peak-WNS	0.30	0.09	3.17	0.003	0.0055
	post-WNS vs pre-WNS	0.54	0.10	5.14	<.0001	<.0001
Arousal temperature Tsk	pre-WNS vs peak-WNS	−2.00	0.90	−2.22	0.0325	0.0445
	post-WNS vs peak-WNS	−1.25	0.84	−1.50	0.1428	0.1714
	post-WNS vs pre-WNS	0.75	0.86	0.87	0.3900	0.3900
Torpor temperature Tsk	pre-WNS vs peak-WNS	−2.05	0.36	−5.68	<.0001	<.0001
	post-WNS vs peak-WNS	−4.00	0.36	−11.26	<.0001	<.0001
	post-WNS vs pre-WNS	−1.95	0.33	−5.87	<.0001	<.0001

Arousal frequency was tested in a GLS model, accounting for differences in residual variance between groups. Arousal duration was tested in a mixed GLM model, accounting for differences in residual variance among individuals in different groups. The multiple comparison *p*-values were FDR-adjusted across the table

**Fig. 2:**
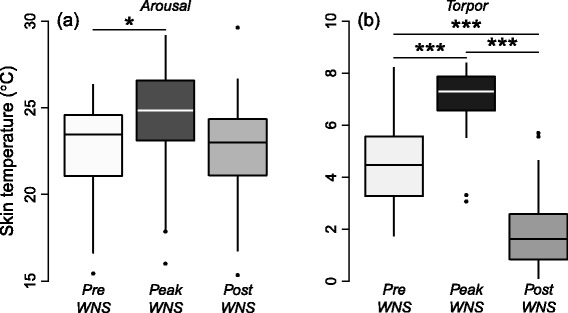
**a** The skin temperature during arousal bouts in in pre-WNS, peak-WNS and post-WNS bats. **b** Torpor skin temperature in pre-WNS, peak-WNS and post-WNS bats. For statistical details, see Table 2. The asterisks ***, **, and * indicate statistical significance of *P* < 0.001, *P* < 0.01, and *P* < 0.05, respectively. Boxes depict the 25th and 75th percentiles, lines within boxes mark the median, and whiskers represent 95th and the 5th percentiles

Post-WNS bats had significantly longer arousal durations than peak-WNS (*p* = 0.0055) bats and pre-WNS bats (*p* < 0.0001) (Table 1, Fig. 1b). Pre-WNS bats had significantly shorter arousal durations than peak-WNS (*p* = 0.045x). Additionally, we found that the arousal T_sk_ of the pre-WNS was significantly lower than peak-WNS bats (Tables 1 & 2). In contrast, arousal T_sk_ did not differ between pre-WNS and post-WNS bats (*p* = 0.3900, Tables 1 and 2, Fig. 2a). Furthermore, all groups differed significantly in their torpor temperature, with post-WNS bats exhibiting the lowest torpor T_sk_ (Tables 1 and 2, Fig. 2b). The analyses of arousal temperatures and arousal durations were not affected by recording frequency of the logger (30 min peak-WNS vs. 10 min pre- and post-WNS) as confirmed by an addition test using every third data point from pre- and post WNS data.

Finally, we wanted to determine if there was a relationship between the extent of *Pd* infection and thermoregulation in the post-WNS group of bats. To verify that the post-WNS bats in NY were infected with *Pd*, we used both qPCR and UV fluorescence. We found that all 22 bats were positive for *Pd* by qPCR in both January and April and exhibited UV fluorescence in April (Additional file 1: Table S1). However, when we compared arousal frequency, arousal duration, arousal T_sk_ and torpor T_sk_ to the level of infection (determined by UV fluorescence) at the end of the hibernation season, we found no significant relationships (data not shown).

## Discussion

Our results indicate that despite being infected with *Pd*, a remnant population of WNS-surviving little brown myotis, hibernating at a site in upstate New York (2015) displayed a significantly lower arousal frequency (and hence longer torpor bouts) than bats dying from WNS in Vermont during the peak mortality phase of the syndrome (2009). Furthermore, arousal frequency and T_sk_ during arousals did not differ between pre- and post-WNS bats. We also found that post-WNS bats had a significantly lower mean torpor T_sk_ compared to either pre- or peak-WNS bats (Fig. 2). However, no difference was found in arousal T_sk_ between peak- and post-WNS bats. Our results show that WNS-survivors appear not only to be showing some pre-WNS hibernation patterns (arousal frequencies), but also more energy-conserving thermoregulation patterns (lower body temperatures during torpor bouts [26]), which may also slow down *Pd* growth in these infected bats [5].

It is unclear if differences in hibernation patterns between pre- and post-WNS bats reflect a shift in the response of the remnant bats to the fungal pathogen or the lack of a response to the pathogen. For example, one plausible explanation for these differences is that WNS survivors were never pre-disposed to the disruptions in torpor patterns described during the peak of mortality [4, 16], and have survived as a result. Bats surviving WNS may not have altered their torpor and arousal behavior or microclimate preferences. Rather, these individuals may represent the small proportion of a pre-WNS population that did not respond to *Pd* infection by increasing arousal frequency, which allowed them to evade WNS-associated mortality. An alternative hypothesis is that shifts in hibernation patterns reflect differences in habitat selection in remnant populations, such as the selection of colder microclimates within hibernacula, which is known to promote longer individual torpor bouts [26].

The hibernaculum that we studied showed no signs of mortality in at the end of winter (April 2015) despite 100 % of the handled bats having signs of WNS. The greater variance in arousal frequency among post-WNS bats, compared to pre-WNS bats could suggest variation in response to *Pd* even among these survivors. The site in NY has been *Pd* positive since at least 2009 (Carl Herzog, pers. comm.). Given the average life expectancy of little brown myotis of approximately 6.5 years [27], it is possible that some of these individuals have dealt with *Pd* every winter since its initial arrival in the area—at least one individual, initially banded in 2010 (Additional file 1: Table S1), was affected for a minimum of five years.

The primary cause of death in species susceptible to WNS is the effect the infection has on the host during torpor, in which bats exhibit an increase in arousal frequency (and thus torpor bout length) [4, 15, 16]. However, post-WNS bats in our study exhibited arousal frequencies that were not statistically different from those in pre-WNS bats and longer arousal durations than either pre- or peak-WNS bats. That said, the slightly raised, although not statistically significant, arousal frequency in the Post-WNS bats compared to Pre-WNS bats could be indicative of a reaction to the ongoing *Pd*-infection, which appears not to be intense enough to cause mortality in this remnant population. Lower ambient temperatures should also result in a lower arousal frequency [28], however no such association was found here using the entire data set, minimizing site effect concerns. Nevertheless, a lower arousal frequency should result in the bat using less stored fat during the winter hibernation period [22]. Unfortunately, we were not able to directly compare the body mass indices of the bats used for this study, as the attachment date of the logger varied between sites and years. Nevertheless, the body masses of post-WNS bats in January were very similar to the pre- and peak-WNS bats from November, suggesting they may have entered hibernation with more fat deposits (see S2). Body mass index may influence torpor bout length and the ability of the bat to withstand *Pd* -infection, but previous results on this are inconclusive [16, 17].

Longer arousal bout duration, more frequent arousals, and higher arousal temperatures are expected to increase the ability of the host to respond to the infection. These host responses may include behavioral responses, such as intensified grooming while euthermic, not allowing the euthermic rest necessary for hibernators [25], and immune responses, such as inflammation [14] and the generation of antibodies to *Pd* [29]. While the generation of an antibody response to *Pd* does not appear to be protective [29], other immune responses may be able to protect bats from *Pd* infection [14]. Hibernation likely suppresses the generation of some cell mediated response, as has been seen in European ground squirrels [10]. However, innate responses to fungal infections may be occurring during periodic arousals or even during torpor [14], although there is no evidence of neutrophil recruitment to sites of *Pd* infection until emergence from hibernation [30]. Another potential benefit of extended arousal duration might be that *Pd* cannot grow at the higher body temperature, especially in the presence of other skin microbiome, which may also be activated by the elevated temperature [31]. Here we found no association between arousal frequency, arousal duration and T_sk_, torpor T_sk_ and *Pd* infection intensity in the post-WNS group at the time of temperature logger removal. Nevertheless, the post-WNS bats appear to be using an energy saving torpor strategy allowing them to tolerate the *Pd*-infection until emergence, when a more effective immune response can occur.

The mechanisms underlying the apparent changes in hibernation patterns could be genetic or behavioral, and could be expressed through phenotypic plasticity. Increased survivorship in *Pd-*affected species like little brown myotis likely depends upon a normal balance between pre-hibernation energy storage and the amount of energy expended during hibernation. There are three inter-related mechanisms that could help restore this balance: 1) bats could enter hibernation with more fat and afford more arousals, or, as presented here, 2) bats could arouse less during hibernation to save fat, and 3) hibernate at lower temperatures to increase energy conservation [32] and slow *Pd* growth [33]. The microclimate a bat hibernates in is critical to the successful completion of a hibernation period [34] because of their thermoconforming nature (but see [35]). The use of a hibernacula, or microclimate within the hibernacula that is below the temperature optimum of *Pd* may allow the bat to evade a persistent and lethal infection. A colder microclimate, to a certain point, also allows for longer hibernation bouts and lower energy expenditure [26], but comes at the expense of expending more energy to arouse from torpor. Both Johnson et al. [17] and Grieneisen et al. [36], under controlled conditions, and Langwig et al. [12] under field conditions, demonstrated a significant, enhanced survival at lower temperatures in *Pd* affected bats. In the current study, we found that post-WNS bats demonstrated the lowest torpor T_sk_ (1.9 ± 1.4 °C). Whether individual bats have altered their behavior to seek out colder microclimates or what we are seeing is the result of selection cannot be distinguished with the data available. Regarding selection, one can speculate that a portion of the historic population has preferred colder microclimates in hibernacula, or used entire hibernacula that are not suitable for *Pd* growth and that influence torpor behavior. However, our data does not allow us to separate evolutionary or physiological explanations and bat populations in northeastern North America are likely still adapting to *Pd*.

## Conclusions

Our results suggest that little brown myotis populations in the eastern United States have adapted to the severe threat posed by WNS in ways that may support persistence of a remnant population over time. We identified three behavioral population level adaptations that may contribute to survivorship: 1) return to a typical Pre-WNS frequency of arousals from torpor, 2) altered duration of arousals, and 3) selection of/benefitting from colder microclimates for torpor. In addition to these factors, survival may be favored by increases in fat reserves prior to hibernation. We predict that little brown myotis may adapt to endemic WNS by exhibiting changes in their thermoregulatory behaviors and by selecting/benefitting from cave microclimates that are less hospitable to the fungal pathogen.

## Methods

This study was carried out on bats from a non-endangered species in strict accordance with the recommendations in the Guide for the Care and Use of Laboratory Animals of the National Institutes of Health. All methods were approved by the Institutional Animal Care and Use Committee at Bucknell University (protocol DMR-016).

### Bat capture protocol and analyses

We used three temporally and geographically discrete data sets from little brown myotis for our analyses: 1) Bats sampled from a mine in southeastern Pennsylvania (Nov 2009 – March 2010, *N* = 9, Nov 2010 – March 2011, *N* = 2) before *Pd* had entered the site (“pre-WNS”), 2) *Pd*-infected bats dying from WNS from a cave in Vermont (Nov 2008 – March 2009, “peak-WNS”, *N* = 12) and 3) *Pd*-infected WNS-survivors from a cave in New York (Jan 2015 – Apr 2015, “post-WNS”, *N* = 19, see Additional file 1: Table S1). While our data are not longitudinal and were collected across a large geographic area, bats from these three locations are expected to belong to the same genetic population [37]. Temperature minimums and maximums within the hibernacula are not known, but all are historic bat hibernacula, thus likely optimal for little brown myotis hibernation [28], with average recorded temperatures of 2.6 °C ±0.5 (PA), 7.2 °C ±4.8 (VT) and 2.6 °C ±2.3 (NY) within a single season from one location in the each of the hibernacula. Means presented are for the hibernation period (November-April). The sampling procedures for the pre-WNS and peak-WNS bats are previously described, with skin temperature (T_sk_) recorded every 10 and 30 min respectively [16]. All data loggers used were calibrated before use.

For the post-WNS data, we captured 50 hibernating little brown myotis from the study site in Washington County, New York on January 30, 2015. The site was officially declared *Pd*-positive in 2009, which was followed by a steep decline in numbers of bats during the next two years. In 2012, roughly 10 % of the original hibernating population remained. Since this time, no dead bats have been found at the site (Carl Herzog, NYDEC, pers. comm.). We sexed, weighed, banded, and measured the forearms of the captured bats. We swabbed the wings with dry cotton swabs (Puritan 25-806 1PD, Guildford ME, USA) for quantitative PCR (qPCR) analysis of *Pd* loads. Subsequently, a small area of hair between the shoulder blades was cut short and a temperature logger (WeeTag, Alpha Mach Inc, Ste-Julie QC, Canada) was attached with liquid bonding cement (Torbot, Cranston RI, USA). The loggers were programmed to record T_sk_ every 10 min.

On April 4, 2015, we recaptured 22 of 50 bats with loggers attached. No dead bats were observed on site. The loggers were carefully removed using a solvent (Dermasol surgical solvent, Perma-Type Inc., Plainville CT) and the bats were again weighed, swabbed for *Pd*, and the wings were photographed with long-wave UV transillumination to assess the infection intensity of *Pd* [38]. The bats were returned to the hibernaculum after handling. Data from the still operational loggers (*n* = 19) were downloaded using the Alpha Mach Inc. Weedot-programming software. Thermal data for all three bat groups available as Additional file 2. Quantification of *Pd* load by qPCR was completed as described previously [29] with the exception of using 1 μl sample in the reaction, Roche Fast Start Essential DNA Probe Master, and a Roche Lightcycler Real Time PCR instead of a BioRad iCycler.

The digital UV images, blinded as to whether the image was from an infected or uninfected bat, were analyzed using Cellprofiler 2.1.1 (Broad Institute). After selecting the green RGB channel, a Wacom Intuos 5 tablet was used to manually trace the border of the wing that was visible (the tip was held down by a finger for the photograph) and in focus within the image. Within the traced wing boundary, *Pd* colonies were identified automatically using the IdentifyPrimaryObjects module with a manual brightness threshold that was adjusted per wing as needed (minimum threshold used: 0.06, maximum threshold used: 0.10) to capture the *Pd* colonies while avoiding recognition of any anomalous brightness that was not *Pd*. Results are presented as the ratio of area of *Pd* to the total wing area.

All pre-WNS bats from Pennsylvania were either negative for *Pd* by histology (*n* = 9) or by PCR (*n* = 2; [33]). The infected bats from Vermont, as far could be ascertained from decaying remains, were determined to be *Pd*-positive by histology [16]. The bats from New York screened PCR positive both at the point of temperature logger attachment and retrieval. These bats also showed clear signs (orange fluorescence) of *Pd* infection under UV transillumination.

### Statistical analysis

**Fig. 3:**
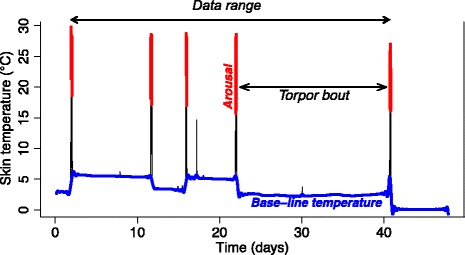
Example of a sampling period with an illustration of the shifts in baseline temperature from which a 10 °C increase is regarded as an arousal. Time between arousals is used to calculate the torpor bout length

All data analysis was performed in R v3.2.0. We defined the baseline skin temperature (*T*_*sk*_) for each individual at each time point by fitting a smoothing spline to temperature values not exceeding 10 °C (Fig. 3). Arousal from hibernation was then determined as a minimum of 10 °C increase from this baseline, lasting at least 20 min. For all calculations, the first (after logger attachment) and last (before logger detachment) periods of torpor were discarded because the bats were handled during these periods. We used *T*_*sk*_ data beginning after the first arousal post logger attachment (i.e. the second torpor bout) and terminated at the end of the last true arousal before logger detachment for our analysis. This removes the first arousal, and the first and last torpor bouts from the data, which we considered artefactual data. The proportional requency of arousals [4] was calculated as the number of arousals divided by the length of the trimmed observation period in days. Arousal frequency was calculated as a single value for each bat. Arousal bout duration was calculated as the duration of each arousal period in minutes after a minimum of 10 °C increase from the baseline temperature. Data are presented as means ± standard deviation.

To determine whether arousal frequency differed between groups (pre-WNS, peak-WNS, post-WNS), we fitted a generalized least squares model (function ‘gls’ in package ‘nlme’), assuming differential residual variance between groups (weights = varIdent(form = ~1|group)). Duration of arousals was analyzed with a GLM model. When analyzing variation in body temperature during arousals, a Gaussian error distribution was assumed instead. We also calculated the arousal temperatures and arousal durations using every third data point in pre- and post- WNS data to account for the 30 min data points in peak WNS to test whether our results would be affected by the data collection frequency of the logger during the relatively short arousals in bats; otherwise data were not normalized between groups for the analyses. Ambient temperature recordings were not used for comparison between groups. Hibernacula are thermally variable environments, but temperatures within hibernacula vary, with numerous microenvironments present. Bats utilize these differences in temperature to their advantage during the hibernation period, i.e. bats relocate to microclimates that suit their energetics at any particular time during the winter [26]. Therefore, we used torpor T_sk_ as a proxy for T_a_ in our statistical model. Significance levels for all multiple comparisons were corrected with false discovery rate (FDR) correction and, unless otherwise noted, these values are presented in the text. Although our statistical model took into account the co-dependence of the samples in order to avoid problems caused by pseudoreplication, we acknowledge the possibility of site effects (such as genetic differences between the bats and differences in the temperature). Finally, we used a linear model for the analysis of the relationship between *Pd* infection intensity and covariates associated with hibernation in the post-WNS group (time since last arousal, arousal frequency, scaled mass index (mass(g))*(38.14/(forearm length(mm))^0.5182 [39]), sex, duration of last arousal bout).

## Additional files

Additional file 1: Table S1. Non-thermal data for individual bats used for this study. (DOCX 112 kb) Additional file 2. Full thermal dataset for all individual bats used for study.

## References

[1] Coleman JTH, Reichard JD (2014). Bat white-nose syndrome in 2014: a brief assessment seven years after discovery of a virulent fungal pathogen in North America. Outlooks Pest Manag.

[2] Minnis AM, Lindner DL (2013). Phylogenetic evaluation of *Geomyces* and allies reveals no close relatives of *Pseudogymnoascus destructans*, comb. nov., in bat hibernacula of eastern North America. Fungal Biol.

[3] Lorch JM, Meteyer CU, Behr MJ, Boyles JG, Cryan PM, Hicks AC (2011). Experimental infection of bats with *Geomyces destructans* causes white-nose syndrome. Nature.

[4] Warnecke L, Turner JM, Bollinger TK, Lorch JM, Misra V, Cryan PM (2012). Inoculation of bats with European *Geomyces destructans* supports the novel pathogen hypothesis for the origin of white-nose syndrome. Proc Natl Acad Sci U S A.

[5] Verant ML, Boyles JG, Waldrep W, Wibbelt G, Blehert DS (2012). Temperature-dependent growth of *Geomyces destructans*, the fungus that causes bat white-nose syndrome. PLoS ONE.

[6] Frick WF, Pollock JF, Hicks AC, Langwig KE, Reynolds DS, Turner GG (2010). An Emerging disease causes regional population collapse of a common North American bat species. Science.

[7] Frick WF, Puechmaille SJ, Hoyt JR, Nickel BA, Langwig KE, Foster JT (2015). Disease alters macroecological patterns of North American bats. Glob Ecol Biogeogr.

[8] Reichard JD, Fuller NW, Bennett AB, Darling SR, Moore MS, Langwig KE (2014). Interannual survival of *Myotis lucifugus* (Chiroptera: Vespertilionidae) near the epicenter of white-nose syndrome. Northeast Nat.

[9] Carey HV, Andrews MT, Martin SL (2003). Mammalian hibernation: cellular and molecular responses to depressed metabolism and low temperature. Physiol Rev.

[10] Prendergast BJ, Freeman DA, Zucker I, Nelson RJ (2002). Periodic arousal from hibernation is necessary for initiation of immune responses in ground squirrels. Am J Physiol-Regul Integr Comp Physiol.

[11] Bouma HR, Carey HV, Kroese FGM (2010). Hibernation: the immune system at rest?. J Leukoc Biol.

[12] Langwig KE, Frick WF, Bried JT, Hicks AC, Kunz TH, Kilpatrick AM (2012). Sociality, density-dependence and microclimates determine the persistence of populations suffering from a novel fungal disease, white-nose syndrome. Ecol Lett.

[13] Meteyer CU, Buckles EL, Blehert DS, Hicks AC, Green DE, Shearn-Bochsler V (2009). Histopathologic criteria to confirm white-nose syndrome in bats. J Vet Diagn Invest.

[14] Field K, Johnson J, Lilley T, Reeder S, Rogers E, Behr M (2015). The white-nose syndrome transcriptome: activation of anti-fungal host responses in wing tissue of hibernating bats. Plos Pathog.

[15] Verant ML, Meteyer CU, Speakman JR, Cryan PM, Lorch JM, Blehert DS (2014). White-nose syndrome initiates a cascade of physiologic disturbances in the hibernating bat host. BMC Physiol.

[16] Reeder DM, Frank CL, Turner GG, Meteyer CU, Kurta A, Britzke ER (2012). Frequent arousal from hibernation linked to severity of infection and mortality in bats with white-nose syndrome. PLoS ONE.

[17] Johnson JS, Reeder DM, McMichael JW, Meierhofer MB, Stern DWF, Lumadue SS (2014). Host, pathogen, and environmental characteristics predict white-nose syndrome mortality in captive little brown myotis (*Myotis lucifugus*). PLoS ONE.

[18] Turner JM, Warnecke L, Wilcox A, Baloun D, Bollinger TK, Misra V (2015). Conspecific disturbance contributes to altered hibernation patterns in bats with white-nose syndrome. Physiol Behav.

[19] Jonasson KA, Willis CKR (2012). Hibernation energetics of free-ranging little brown bats. J Exp Biol.

[20] Boyles JG, Dunbar MB, Whitaker JO (2006). Activity following arousal in winter in North American vespertilionid bats. Mammal Rev.

[21] Geiser F (2013). Hibernation. Curr Biol.

[22] Thomas D, Cloutier D, Gagne D (1990). Arrhythmic breathing, apnea and non-steady-state oxygen-uptake in hibernating little brown bats (*Myotis lucifugus*). J Exp Biol.

[23] Thomas D, Cloutier D (1992). Evaporative water-loss by hibernating little brown bats, *Myotis lucifugus*. Physiol Zool.

[24] Daan S, Barnes BM, Strijkstra AM (1991). Warming up for sleep? Ground squirrels sleep during arousals from hibernation. Neurosci Lett.

[25] Brownlee-Bouboulis SA, Reeder DM (2013). White-nose syndrome-affected little brown myotis (*Myotis lucifugus*) increase grooming and other active behaviors during arousals from hibernation. J Wildl Dis.

[26] Boyles JG, Dunbar MB, Storm JJ, Brack V (2007). Energy availability influences microclimate selection of hibernating bats. J Exp Biol.

[27] Keen R, Hitchcock H (1980). Survival and Longevity of the Little Brown Bat (*Myotis lucifugus*) in Southeastern Ontario. J Mammal.

[28] Humphries MM, Thomas DW, Speakman JR (2002). Climate-mediated energetic constraints on the distribution of hibernating mammals. Nature.

[29] Johnson JS, Reeder DM, Lilley TM, Czirják GÁ, Voigt CC, McMichael JW (2015). Antibodies to *Pseudogymnoascus destructans* are not sufficient for protection against white-nose syndrome. Ecol Evol.

[30] Meteyer CU, Barber D, Mandl JN (2012). Pathology in euthermic bats with white nose syndrome suggests a natural manifestation of immune reconstitution inflammatory syndrome. Virulence.

[31] Hoyt JR, Langwig KE, Okoniewski J, Frick WF, Stone WB, Kilpatrick AM (2015). Long-term persistence of *Pseudogymnoascus destructans*, the causative agent of white-nose syndrome, in the absence of bats. EcoHealth.

[32] Humphries MM, Speakman JR, Thomas DW, Zubaid A, McCracken GF, Kunz TH (2005). Temperature, hibernation energetics, and the cave and continental distributions of little brown myotis. Functional and evolutionary ecology of bats.

[33] Cryan PM, Meteyer CU, Blehert DS, Lorch JM, Reeder DM, Turner GG (2013). Electrolyte depletion in white-nose syndrome bats. J Wildl Dis.

[34] Kokurewicz T (2004). Sex and age related habitat selection and mass dynamics of Daubenton’s bats *Myotis daubentonii* (Kuhl, 1817) hibernating in natural conditions. Acta Chiropterologica.

[35] Willis CKR, Lane JE, Liknes ET, Swanson DL, Brigham RM (2005). Thermal energetics of female big brown bats (*Eptesicus fuscus*). Can J Zool-Rev Can Zool.

[36] Grieneisen LE, Brownlee-Bouboulis SA, Johnson JS, Reeder DM (2015). Sex and hibernaculum temperature predict survivorship in white-nose syndrome affected little brown myotis (*Myotis lucifugus*). R Soc Open Sci.

[37] Vonhof MJ, Russell AL, Miller-Butterworth CM (2015). Range-wide genetic analysis of little brown bat (*Myotis lucifugus*) populations: estimating the risk of spread of White-nose syndrome. PLoS ONE.

[38] Turner GG, Meteyer CU, Barton H, Gumbs JF, Reeder DM, Overton B (2014). Nonlethal screening of bat-wing skin with the use of ultraviolet fluorescence to detect lesions indicative of white-nose syndrome. J Wildl Dis.

[39] Peig J, Green AJ (2009). New perspectives for estimating body condition from mass/length data: the scaled mass index as an alternative method. Oikos.

