# Supplementary material for: White-nose syndrome survivors do not exhibit frequent arousals associated with Pseudogymnoascus destructans infection
Source: Front Zool. 2016 Mar 3;13:12. doi: 10.1186/s12983-016-0143-3 (PMC4778317; doi:10.1186/s12983-016-0143-3)
Supplement: Additional file 1: Table S1. — Non-thermal data for individual bats used for this study. (DOCX 112 kb) [file 12983_2016_143_MOESM1_ESM.docx]

Table S1. Data on individual bats used for study.

| Bat ID | Study site | Year of banding | Sex | Weight J (g) | Weight A(g) | Forearm | Start date | End date | Jan qPCR | April qPCR | Pd confirmation | Pd status | qUV |
| --- | --- | --- | --- | --- | --- | --- | --- | --- | --- | --- | --- | --- | --- |
| DMR01677 | NY | 2015 | F | 7.71 | 6.69 | 38.08 | 31.1.2015 | 4.4.2015 | 1207 | 23645 | qPCR, UV | + | 16.99 |
| DMR01678 | NY | 2015 | M | 8.47 | 6.86 | 37.72 | 31.1.2015 | 4.4.2015 | 67077 | 357429 | qPCR, UV | + | 2.67 |
| NYDEC65559 | NY | 2014 | M | 9.07 | 8.05 | 39.96 | 31.1.2015 | 4.4.2015 | 143735 | 7428 | qPCR, UV | + | 13.99 |
| NYDEC4898 | NY | 2014 | M | 8.73 | 6.86 | 38.26 | 31.1.2015 | 4.4.2015 | 19343 | 6982 | qPCR, UV | + | 21.65 |
| NYDEC31845 | NY | 2010 | M | 8.6 | 7.11 | 35.7 | 31.1.2015 | 4.4.2015 | 158489 | 11068 | qPCR, UV | + | 10.01 |
| NYDEC65600 | NY | 2013 | M | 9.42 | 7.61 | 39.76 | 31.1.2015 | 4.4.2015 | 114082 | 806 | qPCR, UV | + | 3.3 |
| DMR01683 | NY | 2015 | M | 9.79 | 7.77 | 40 | 31.1.2015 | 4.4.2015 | 866 | 5333 | qPCR, UV | + | 14.01 |
| DMR01689 | NY | 2015 | M | 7.94 | 6.93 | 36.54 | 31.1.2015 | 4.4.2015 | 14713 | 35708 | qPCR, UV | + | 8.79 |
| DMR01686 | NY | 2015 | M | 8.05 | 6.58 | 36.84 | 31.1.2015 | 4.4.2015 | 4409 | 14893 | qPCR, UV | + | 15.95 |
| DMR01687 | NY | 2015 | F | 7.72 | 6.75 | 39.84 | 31.1.2015 | 4.4.2015 | 33515 | 2559 | qPCR, UV | + | 28.09 |
| DMR01688 | NY | 2015 | M | 7.25 | 6.52 | 35.62 | 31.1.2015 | 4.4.2015 | 57615 | 53657 | qPCR, UV | + | 9.21 |
| DMR01690 | NY | 2015 | M | 8.09 | 7.39 | 40.2 | 31.1.2015 | 4.4.2015 | 282241 | 1048 | qPCR, UV | + | 25.81 |
| OOO2992 | NY | 2012 | M | 7.63 | 6.6 | 36.6 | 31.1.2015 | 4.4.2015 | 42571 | 1710 | qPCR, UV | + | 12.06 |
| DMR01695 | NY | 2015 | M | 7.45 | 6.83 | 37.68 | 31.1.2015 | 4.4.2015 | 12578 | 42062 | qPCR, UV | + | 8.62 |
| DMR01698 | NY | 2015 | M | 8.3 | 7.82 | 39.77 | 31.1.2015 | 4.4.2015 | 2871 | 193188 | qPCR, UV | + | 0.2 |
| DMR02467 | NY | 2015 | F | 7.37 | 6.99 | 37.53 | 31.1.2015 | 4.4.2015 | 6963 | 19647 | qPCR, UV | + | 7.47 |
| NYDEC65539 | NY | 2014 | M | 7.04 | 6.55 | 37.53 | 31.1.2015 | 4.4.2015 | 69589 | 5469 | qPCR, UV | + | 21.64 |
| DMR02469 | NY | 2015 | F | 8.65 | 7.86 | 40.66 | 31.1.2015 | 4.4.2015 | 211 | 5082 | qPCR, UV | + | 16.93 |
| DMR02470 | NY | 2015 | M | 8.58 | 6.58 | 38.14 | 31.1.2015 | 4.4.2015 | 40407 | 47937 | qPCR, UV | + | 6.58 |
| AAA341 | NY | 2013 | M | 8.05 | 7.28 | 37.54 | 31.1.2015 | 4.4.2015 | 108916 | 36921 | qPCR, UV | + | N/A |
| DMR02475 | NY | 2015 | M | 8.09 | 6.68 | 37.06 | 31.1.2015 | 4.4.2015 | 9755 | 5108 | qPCR, UV | + | 19.55 |
| DMR02476 | NY | 2015 | F | 8.58 | 7.17 | 39.4 | 31.1.2015 | 4.4.2015 | 326 | 15594 | qPCR, UV | + | 10.08 |
| VT5 | VT | N/A | M | 7.4 | N/A | 39.8 | 14.11.2008 | 25.2.2009 | N/A | N/A | Histology | + | N/A |
| VT10n | VT | N/A | F | 9 | N/A | 37.3 | 14.11.2008 | 25.2.2009 | N/A | N/A | Histology | + | N/A |
| VT18n | VT | N/A | M | 7.4 | N/A | 39 | 14.11.2008 | 25.2.2009 | N/A | N/A | Histology | + | N/A |
| VT21 | VT | N/A | M | 8.1 | N/A | 37.6 | 14.11.2008 | 25.2.2009 | N/A | N/A | Histology | + | N/A |
| VT32 | VT | N/A | F | 7.9 | N/A | 38.7 | 14.11.2008 | 25.2.2009 | N/A | N/A | Histology | + | N/A |
| VT36 | VT | N/A | F | 8.3 | N/A | 38.5 | 14.11.2008 | 25.2.2009 | N/A | N/A | Histology | + | N/A |
| VT52 | VT | N/A | M | 7.7 | N/A | 38.7 | 14.11.2008 | 25.2.2009 | N/A | N/A | Histology | + | N/A |
| VT62 | VT | N/A | F | 8 | N/A | 37.2 | 14.11.2008 | 25.2.2009 | N/A | N/A | Histology | + | N/A |
| VT-003 | VT | N/A | F | 7.7 | N/A | 38 | 14.11.2008 | 25.2.2009 | N/A | N/A | Histology | + | N/A |
| VT-004 | VT | N/A | M | 7.4 | N/A | 37.4 | 14.11.2008 | 25.2.2009 | N/A | N/A | Histology | + | N/A |
| VT-005 | VT | N/A | M | 8.4 | N/A | 37.7 | 14.11.2008 | 25.2.2009 | N/A | N/A | Histology | + | N/A |
| VT-030 | VT | N/A | M | 8.4 | N/A | 37.7 | 14.11.2008 | 25.2.2009 | N/A | N/A | Histology | + | N/A |
| LAYT46 | PA | N/A | F | 10.24 | N/A | 38 | 14.11.2009 | 25.3.2010 | N/A | N/A | Histology | - | N/A |
| LAYT53 | PA | N/A | F | 10.22 | N/A | 39 | 14.11.2009 | 25.3.2010 | N/A | N/A | Histology | - | N/A |
| LAYT17 | PA | N/A | F | 9.45 | N/A | 41 | 14.11.2009 | 25.3.2010 | N/A | N/A | Histology | - | N/A |
| LAYT28 | PA | N/A | M | 8.53 | N/A | 39 | 14.11.2009 | 25.3.2010 | N/A | N/A | Histology | - | N/A |
| LAYT29 | PA | N/A | M | 7.85 | N/A | 36 | 14.11.2009 | 25.3.2010 | N/A | N/A | Histology | - | N/A |
| LAYT1 | PA | N/A | F | 8.13 | N/A | 38.5 | 14.11.2009 | 25.3.2010 | N/A | N/A | Histology | - | N/A |
| LAYR30 | PA | N/A | M | 7.13 | N/A | 35.5 | 14.11.2009 | 25.3.2010 | N/A | N/A | Histology | - | N/A |
| LAYR71 | PA | N/A | M | 7.33 | N/A | 37 | 14.11.2009 | 25.3.2010 | N/A | N/A | Histology | - | N/A |
| LAYT5 | PA | N/A | F | 7.97 | N/A | 37 | 14.11.2009 | 25.3.2010 | N/A | N/A | Histology | - | N/A |
| LAY314 | PA | N/A | M | 7.6 | N/A | 37 | 19.10.2010 | 10.3.2011 | N/A | N/A | qPCR | - | N/A |
| LAY339 | PA | N/A | F | 8.5 | N/A | 36 | 19.10.2010 | 10.3.2011 | N/A | N/A | qPCR | - | N/A |
